# The experience of transitions in care in very old age: implications for general practice

**DOI:** 10.1093/fampra/cmz014

**Published:** 2019-05-04

**Authors:** Fiona Scheibl, Jane Fleming, Jackie Buck, Stephen Barclay, Carol Brayne, Morag Farquhar

**Affiliations:** 1 Department of Public Health and Primary Care, Cambridge Institute of Public Health, University of Cambridge, Cambridge, UK; 2 School of Health Sciences, University of East Anglia, Norwich, UK

**Keywords:** Care homes, falls, frailty, general practice, oldest old, relocation stress syndrome

## Abstract

**Background:**

It can be challenging for general practitioners to support their oldest old patients through the complex process of relocation.

**Objective:**

To provide a typology of the experiences of moving in very old age that is clinically useful for practitioners navigating very old people’s relocation.

**Methods:**

Qualitative analysis of data from a mixed-methods UK population-based longitudinal study, Cambridge City over-75s Cohort (CC75C), from Year 21 follow-up onwards. Interviews with participants aged ≥95 years old and proxy informants (Year 21: 44/48, 92%, subsequent attrition all deaths). Thematic analysis of qualitative data available from 26/32 participants who moved before they died.

**Results:**

Individuals who moved voluntarily in with family experienced gratitude, and those who moved into sheltered house or care homes voluntarily had no regrets. One voluntary move into care was experienced with regret, loss and increased isolation as it severed life-long community ties. Regret and loss were key experiences for those making involuntary moves into care, but acceptance, relief and appreciation of increased company were also observed. The key experience of family members was trauma. Establishing connections with people or place ahead of moving, for example through previous respite care, eased moving. A checklist for practitioners based on the resulting typology of relocation is proposed.

**Conclusions:**

Most of the sample moved into residential care. This study highlights the importance of connections to locality, people and place along with good family relationships as the key facilitators of a healthy transition into care for the oldest old. The proposed checklist may have clinical utility.

**Key Messages**

- Resilience enables very old people to adapt following relocation.
- Some very old people prefer having company in residential care to living alone.
- Moves that sever life-long local connections increase isolation and loss.
- Establishing connections with people or place ahead of a move is beneficial.

## Introduction

As the population ages, general practitioners (GPs) are increasingly called upon to support their oldest old patients though the challenges of frailty/multi-morbidity and relocation. The oldest old with moderate levels of disability are likely to make voluntary assistance seeking moves to be closer to relatives after a trigger such as bereavement (1, 2). The likelihood of making an involuntary move into long-term residential or nursing care settings increases in very old age (1, 3, 4) prompted by frailty, falls and hospitalization (5).

Relocation can be stressful for older people and most will experience anxiety, some confusion, others depression—a process conceptualized as ‘Relocation Stress Syndrome’ (RSS) (6–8). Because the oldest old are likely to make multiple transitions (from hospital to respite care and to more than one care home), the risk of RSS increases (2, 9, 10). Transition theory defines the conditions needed to make healthy transitions as (i) personal resilience, (ii) support from family or community and (iii) societal conditions that are supportive of older people (good-quality residential care) (11, 12). Some degree of ownership of the decision to move is also required for the transition to be healthy (2). Ownership is particularly important for people living with dementia because they have lower levels of resilience and hold both positive and negative views on moving into care (‘having someone to care for me’/‘not being able to go out’ (13). Language is also important: individuals should not be described as being ‘placed’ in a care home, which implies passivity, but seen as ‘living’ in the home (2, 14, 15). Although individuals are more likely to make a healthy transition if they are resilient and ‘buy into’ the decision to move, the onus is on care home providers to cultivate a sense of ‘home’, and present a ‘welcoming’ workforce culture for new residents (16).

Persons aged ≥85 are much more likely to consult their GP (17) and it is likely that they will be the first point of call when a health care crisis triggers the need for relocation. However, the sustainability of the patient–doctor relationship is challenged once the person moves into residential care as contact becomes variable ranging from episodic and reactive, to integral relationships that facilitate high-quality person-centred care (18). GPs’ attitudes towards care home residents vary; some feel burdened or helpless when confronted by the severity of illness among this population (19), whereas others find their role rewarding and meaningful (20). In this study, we draw on qualitative data from a unique sample aged ≥95 to identify the experiences of moving among the oldest old and devise a proposed checklist which may help practitioners respond to, or avert, individual’s anxiety about relocation.

## Methods

### Study design

The CC75C study’s methods have been previously described, both for the cohort overall (21) and for the qualitative component (22). Briefly, the original population-based sample (*n* = 2166, 95% response rate), enrolled in 1985/87 using general practice lists, were re-interviewed every few years until 2013 (Survey 10: Year 28, the final survey before all participants had died). Each survey wave, which included assessments of cognition (23, 24) and disability (25), obtained Cambridge Research Ethics Committee approval and renewed consent. At Year 21 surviving participants were invited to an additional interview with the aim of understanding ‘what it is like to be so old’ which included exploring experiences of moving (26). Proxies, usually relatives, were also interviewed to gather their insights into these very old peoples’ experiences. Interviews with participants and proxies were conducted in their usual residence, audio-recorded, and transcribed. All data were anonymized and identifying characteristics removed. Where quotes are presented in the text below pseudonyms are used to maintain anonymity. The cognitive status of participants is indicated by the abbreviations ‘SCI’ denoting severe impartment, ‘ModCI’ moderate impairment, ‘MCI’ mild impairment, and ‘NCI’ not impaired.

### Data analysis

Descriptive statistics used quantitative data from core survey measures to summarize sample characteristics. We used study archives and databases to track address changes to identify transitions to different residential settings. The qualitative data were analysed thematically working from primary coding to more complex themes and connections as the data were charted into a framework matrix using NVivo qualitative data analysis software.

## Results

### Moving residence in very old age

**Figure 1.**
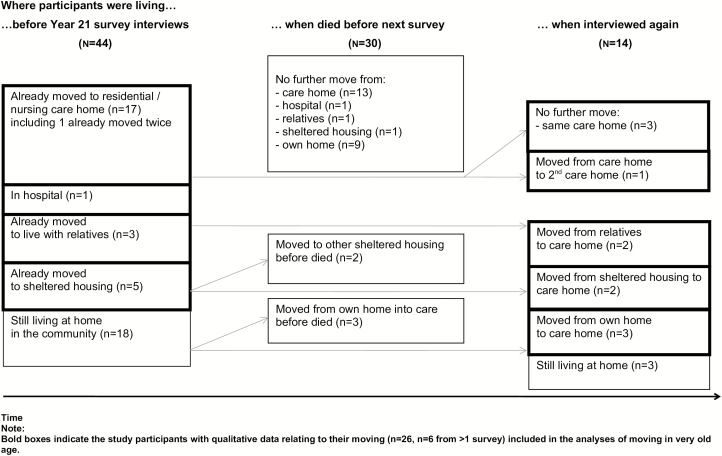
This illustrates where participants had moved to before the qualitative interviews took place in Year 21 and subsequently. Of the participants who had already moved 29% (8/26) later moved again. Moves before this survey were on average several years before, median 3.8 years (IQR: 1.3–5.9), and moves for the remainder who subsequently moved were a median 2.3 years (IQR: 1.2–3.5) later. Two-thirds died before any later survey (30/44, 68%).

CC75C’s Year 21 (2006–07) survey included 44 of 48 surviving participants (92%). Fewer than half still lived in their own home (18/44, 41%), dropping to just over a quarter by the time they died (12/44, 27%). Figure 1 illustrates where participants had moved to before this survey and subsequently. Of those who had already moved, 29% (8/26) later moved again. Moves before this survey were several years previously, median 3.8 years (IQR: 1.3–5.9); moves for those who moved again were a median 2.3 years (IQR: 1.2–3.5) later. Two-thirds died before any later survey (30/44, 68%), some with no interview discussion of moving, but relevant qualitative data were available for the majority of participants who moved at any point (26/32, 80% of those who moved): the sample included in this analysis. Five of the 26 had moved before 2006, 15 moved during 2007, and six who moved during 2008–10 were interviewed in the final survey.

### Characteristics of participants

Table 1 describes the Year 21 survey participants showing that the prevalence of demographic characteristics, cognitive impairment and disability did not differ greatly between those who moved (*n* = 32) and those who did not (*n* = 12). No differences reached statistical significance, nor did any difference between the total (*n* = 32) who moved and the sub-group (*n* = 26) in this qualitative data analysis (not shown). These 26 participants were aged 95 to 100 years old, median age 97.3 (IQR: 96–98.3); all but one of them were women, and most had cognitive impairment. Most (81%) needed help with basic Activities of Daily Living (ADLs), two only with instrumental ADLs and three with none. However, for those who moved after this survey, both disability and cognitive impairment had worsened by the time they moved. Supplementary Table S1 provides summary characteristics for each participant, listed in order of quotation, each of whom is allocated a de-personalized pseudonym for ease of identification.

**Table 1.** Characteristics of *n* = 44 CC75C study participants at Year 21 follow-up (2006–07)

	All (*n* = 44)		Moved from own home before death (*n* = 32)		Not moved from own home before death (*n* = 12)		Significance(*p* value)a
Age (years)							0.733
Mean (SD)	97.4 (1.5)		97.4 (1.4)		97.6 (1.8)		
Median (IQR)	97.1 (96.2–98.4)		97.2 (96.0–98.4)		96.9 (96.3–98.8)		
Range	95.4–101.4		95.5–100.4		95.4–101.4		
Age when 1st moved							
Mean (SD)	–		96.3 (2.9)		–		
Median (IQR)	–		97.1 (94.2–98.3)		–		
Range	–		89.8–101.2		–		
	*n*	(%)	*n*	(%)	*n*	(%)	
Sex							0.116
Male	5	(11)	2	(6)	3	(25)	
Female	39	(89)	30	(94)	9	(75)	
Accommodation							<0.001
Private house/flat	21	(48)	9	(28)	12	(100)	
Sheltered housing	5	(10)	5	(16)	0		
Care home	17	(40)	17	(53)	0		
Long stay hospital	1	(2)	1	(3)	0		
Marital status							1.000
Married	3	(7)	2	(6)	1	(8)	
Widowed	38	(86)	27	(84)	11	(92)	
Separated/Divorced	1	(2)	1	(3)	0		
Single	2	(5)	2	(6)	0		
Education (school leaving age)							0.507
<15 years of age	25	(57)	17	(53)	8	(67)	
≥15 years of age	19	(43)	15	(47)	4	(33)	
Social class^b^ (occupation)							0.735
Non-manual	25	(57)	19	(59)	6	(50)	
Manual	19	(43)	13	(41)	6	(50)	
Cognitive function^c^							0.492
Normal cognition	11	(25)	8	(25)	3	(25)	
Mildly impaired	8	(18)	4	(12.5)	4	(33)	
Moderately impaired	10	(23)	8	(25)	2	(25)	
Severely impaired	15	(34)	12	(37.5)	3	(17)	
Disability in ADLs^d^							1.000
No disability	6	(14)	5	(16)	1	(8)	
IADL disability only	6	(14)	4	(13)	2	(17)	
IADL + BADL disability	32	(73)	23	(72)	9	(75)	

Column percentages may not total 100% due to rounding each percentage.

^a^Significance tests: Fisher’s Exact test for differences in proportions of categorical variables and independent-sampe t-test for the categorical variable age.

^b^Social class categorized following contemporary UK Office of National Statistics grading of occupation reported at baseline interview: Non-manual = I, II or IIIa, Manual = IIIb, IV or V.

^c^Mini-Mental State Examination complete scores, plus score category imputation and dementia status if incomplete, categorized 0–17 severe cognitive impairment, 18–21 moderate cognitive impairment, 22–25 mild cognitive impairment and 26–30 normal cognition.

^d^IADL, Instrumental Activities of Daily Living; BADL, Basic (personal) Activities of Daily Living.

### Characteristics of proxies

Qualitative data were gathered from both participants and proxies for the majority (20/26, 77%; see Supplementary Figure S2). For three participants, two proxies were interviewed and most proxies were women (24/29, 83%). Proxies were daughters (*n* = 14), sons (*n* = 5), other relatives (three children-in-law, two nieces and a sister) or a care home manager (*n* = 1).

### Where participants moved to and the types of moves they made

Seven participants with moderate disability who had recently experienced a bereavement or declining health made voluntary ‘assistance seeking’ moves either in with family, to a care home closer to family or to smaller easier to manage sheltered housing in their local community. The remaining 19, most of whom had higher levels of physical and cognitive disability, moved into residential or nursing homes or long stay hospital (*n* = 1) after a health care crisis had enforced a move.

### Thematic typology

We identified four transition pathways among the oldest old and their associated experiences (summarized in Table 2).

**Table 2.** Four transition pathways and their associated experiences—identified from participant and proxy informant interviews with *n* = 26 CC75C study participants aged ≥95 who moved in very old age (Year 21 follow-up: 2006–07)

Voluntary move to proximal sheltered housing or in with family	Involuntary move to proximal residential care	Voluntary move to geographically distant residential care	Involuntary move to second proximal care home
No regrets Gratitude and appreciation	Regret and loss Relief Acceptance and resignation Reduced loneliness	Regret and loss Increased isolation	Instability and trauma Feeling unconnected
Creates conditions for a healthy transition		Does not create conditions for a healthy transition	

### Experiences associated with making a voluntary move

Seven moved voluntarily in with family (*n* = 2), sheltered housing (*n* = 1) or into a care home closer to family (*n* = 4). One had no cognitive impairment, two were moderately impaired and four had severe cognitive impairment. Moves in with family and sheltered housing were experienced with gratitude and improved well-being and met the conditions for a healthy transition:

*Stella Thatcher: Oh, I was very grateful that they (family) would take me here because it was very lonely on my own. (SCI)*

*Rose Baker: When I came here I knew quite a few people. One, for the fact that I used to live across the road years ago [..] And I used to work at the post office up here. […] being able to mix with others makes life so much easier. (NCI)*

Participants viewed moving into care as an altruistic act that protected family from the burden of care:

*Patricia Miller: But you’ve got to be sensible and not put yourself first, you’ve got to the think about the others. That’s the way you should be here. (SCI)*

Participants accepted the need to change, were quite philosophical about the need to move and saw it as part of adapting to the process of ageing:

*Charlotte Smith: You get to the stage […] you take life as it comes. A part of life you have control of, but there comes a time, and that’s in my case, when you don’t have the same control, (ModCI)*

*Patricia Miller: I didn’t want to give up my independence, which to a certain extent you’ve got to. But you’ve got to think about it in the right sort of way. (SCI)*

For one participant who moved to a care home in a different region of the country closer to her daughter the experience was one of isolation:

*Florence Potter: I’ve got nothing to live for. (My daughter and her family) they’ve got their own lives to live and she’s busy. She doesn’t often come [to visit]. ’Cos it was a damn silly thing I did to come here from Cambridge. […] I’ve never really settled. [it’s] Their… tone somehow. Northern… Northerners are like people in a different country almost’. (SCI)*

Relatives were distressed by their older relatives decline in health and need to move: 11 (55%) of the 20 proxies used the word ‘trauma’ to describe the events leading up to relocation:

*Charlotte Smith’s daughter: Mum was [..] very ill […] we brought you back here and you [addressing her mother] couldn’t get out of bed […]. And then [..] you wanted to go back [home]. And then one day we popped in on you and you were [..] sitting on the back doorstep, very sad. And you said “I want to come and live with you.” […..] It was very traumatic really. (ModCI)*

Relatives also acknowledged that the experience of moving into care was distressing and expressed ambivalence about the extent to which older people could settle:

*Prudence Sawyer’s daughter: Oh, she wasn’t happy. And we were told by the people in the home there not to go and see her for two or three weeks. “Let her get settled in.” And she wasn’t happy at all. [..] She’s OK with it now, I think. [..] She’s accepted... accepted it. (SCI)*

### Experiences associated with making an involuntary move into a care home

By far the majority (19/26, 73%) moved into care homes, their experiences reflecting the largely involuntary nature of these moves. Moves were typically triggered by a crisis (injurious fall, hospitalization, incontinence, declining mobility and loss of care) which limited their capacity during the decision making process. All nineteen had some level of cognitive impairment.

Seventeen of these 19 regretted the loss of familiar objects associated with their own homes and some, like the participant quoted below, felt isolated in the monotony of life in care:

*Hyacinth Fletcher: I meet the same old faces. They don’t speak. There’s no conversation. […] They’ve always been like it. [..] And my [kitchen] weights went right the way down to half an ounce. They were worth something. They went. (SCI)*

The lack of freedom and privacy was also an issue:

*Margaret Butcher: I don’t like being here, to be honest. I’d rather have my freedom. […], There’s always somebody bustling round you […] you don’t have much privacy. […] I don’t think you’re allowed to go very far. […] Yes, if it’s only a small walk. […] This is nowhere near... like the comfort of your home. (SCI)*

But difficulties managing at home or with care arrangements meant that making a move into a care home was experienced by some with a sense of relief when care home staff were welcoming, a pre-condition for a healthy transition:

*Nancy Dempster’s daughter: […] They (the care home staff) seemed to understand how it had all been so stressful. [..] they invited her to come and have a look round, timed it so we could stay for lunch, showed her some of the things that were going on. She’s really come alive again since moving there. (MCI)*

Eleven of the 19 who moved into care said that they had accepted or resigned themselves to the transition, acknowledging that achieving great age meant accepting less independence. This finding held for those with cognitive impairment and those without impairment.

*Agatha Cooper Well, I’m quite content. I wouldn’t want my life to be any different. I’m satisfied. I’ve got no worries about anything. (ModCI)*

*Flora Chamberlain’s daughter: It’s a little residential home, only five people, a sherry before lunch, you know that kind of thing. And she got very used to it […] and I think she was fairly resigned and fairly happy. (SCI)*

*Archibald Faulkner: I think you come to accept it. […] …it’s done with the best of intentions. […] if they said “Go back home” I should be appalled. I shouldn’t know what [I’d] be able to do. (NCI)*

The data indicate that acceptance of a new life in care is undermined if the current care home takes steps to relocate a resident to a different home. In these circumstances, the older persons’ stability is lost as they no longer feel connected or supported:

*Flora Chamberlain’s daughter: I have to [..] see if I can get a nurse to be with her while I go because she’s always sort of saying to me “[..], “Don’t go without me.” […] Yes, her phrase when she actually first went in there [the second care home] was “You’ve done for me.” […] and it’s pretty well done for her in that now she is, you know they’ve been sedating her a bit,’ (SCI)*

For nine (47%) of the 19 who moved into care, this was experienced as preferable to living alone:

*Interviewer: What is good about living here?*

*Primrose Turner: To go to the lounge and see some people on television with all the others. I know I’ve got my own [television] here, but it’s not the same as when you’re near others. (ModCI)*

*Interviewer: So what’s it like living here at the moment?*

*Loretta Fowler: I like being here because there’s company. And living alone it is miserable. (It’s) better than staying alone in the house. (SCI)*

## Conclusions

Voluntary moves proximal to their most recent dwelling generated gratitude, appreciation and no regrets. A voluntary move that severed life-long community ties was experienced with regret and increased isolation due to the absence of a good family or community network. Regret and loss were key experiences for those making involuntary moves into care, but there was also relief, acceptance and an appreciation of increased company and reduced loneliness. In the fourth type of move, one individual was required to move involuntarily to a second care home and she struggled to achieve the inner balance typical of a healthy transition. Given that the home care and care home environment has been under extensive pressure since the CC75C interviews were completed in 2006–10, we suggest that further research is needed to consider the incidence of people being moved to another care setting without any choice, and how this affects their well-being.

### Comparison with existing literature

This is the first study to examine transitions in care in the very oldest members of our society. Our data support the view that, if their preferences are overridden, older people may not adjust as well to life in care (14). Our results also confirm that older people narrate their experience of transition with stories of lost objects (27) and this sense of loss extends to home, privacy and activities (28). Some CC75C participants were challenged to feel ‘at home’ in ‘busy’ care homes, echoing previous studies highlighting difficulties feeling at home in a place of work (27,29). Our data reinforce the finding that older people living with dementia had both positive and negative views on moving into care (having someone to care for me versus not being able to go out) (13). In line with previous research, our data suggest that acceptance of life in care is possible when the person is resilient and can adjust to the loss of control over their life (29). Our data also demonstrate how the welcoming nature of the care home has a positive impact (16). Our findings counter the dominant view that life in a care home is a completely negative experience: some of the older old people in this study preferred having the company of others in their care home to living an isolated life alone in their own home.

A key strength of this study is the qualitative data obtained in a cohort study highly representative of the growing ‘oldest old’ population. A methodological limitation of the CC75C data is that it does not permit analytical separation of the effects of moving from the conditions that prompted the move. We also acknowledge the limitations of proxy reports (the only data available for six of the 26 participants), but they provide insight into the experiences of very old age that would otherwise be entirely missing. Another limitation is the possibility of recall bias, as people were interviewed at different time points following their relocation.

### Implications for practice

To aid GPs working in this field, we propose a checklist (Box 1) signposting resources aimed at supporting people in the process of moving and those who have recently relocated who may be experiencing anxiety, confusion, depression and isolation typical of RSS.

The proposed checklist provides a structured framework to stimulate discussion and orientate older people and their families. GPs might work through each of the stages with patients who need to relocate. Or it could be used as an additional prompt when completing advanced care plans or frailty assessments. For example, GPs working with patients in care homes who exhibit signs of RSS or depression could consider allocating a social worker or consider social prescribing of social or physical activity and recommend dietary improvements in collaboration with care home staff. Further research is needed to establish stakeholder endorsement of the proposed checklist and its utility for GPs.

Box 1. **Proposed checklist with resources to support patients through relocation in very old age**

**Before a move into residential care**

✓ Encourage your patients, if possible, and their carers to conduct research before they choose a care home in their current locality to maintain cultural, social and ethnic connections. A checklist of things to consider is available at https://www.which.co.uk/later-life-care/housing-options/care-homes/choosing-a-care-home-azff15m2v43f.
✓ Assess your patient’s capacity for involvement in the decision making. Alzheimer’s UK has a section on decision making and recommends that family should be prepared and have discussions as early as possible, which can help make the decision slightly easier when the time comes:
https://www.alzheimers.org.uk/get-support/daily-living/making-decisions-around-residential-or-nursing-care#content-start and https://www.caba.org.uk/help-and-guides/information/caring-ageing-parents.
✓ Could your patient have a trial stay in respite care? Dementia UK guidance on the process of moving into a care home recommends a trial period as a first step for moving:
https://www.dementiauk.org/advice-on-moving-into-a-care-home/.
✓ If your patient is in hospital apply the ‘Discharge to Assess’ principles before a final placement in residential care is decided upon. This principle is being introduced in the NHS:
https://www.england.nhs.uk/urgent-emergency-care/hospital-to-home/improving-hospital-discharge/discharge-to-assess/.
✓ Allay your patient’s fears about how they will settle in, offering the ‘settling in’ leaflet provided by Age UK which sets out key steps for adjusting to life in care:
https://www.ageuk.org.uk/information-advice/care/arranging-care/care-homes/moving-into-care-home/ and
https://seniorcarecoalition.org/reducing-relocation-stress/.
✓ Support your patient through the initial stages of the move—visit them in their new home to ensure continuity of care. This principle is being instituted in the NHS:
http://www.pulsetoday.co.uk/clinical/clinical-specialties/elderly-care/gps-to-do-weekly-care-home-rounds-under-new-nhs-england-plan/20032921.article.

**During and after moving**

✓ Consider allocating a case worker/social worker/support group intervention to provide extra support to promote meaningful activity and boost well-being: https://www.nice.org.uk/guidance/qs50/chapter/Development-sources;
https://www.rcgp.org.uk/clinical-and-research/resources/toolkits/mental-health-toolkit.aspx;
https://seniorcarecoalition.org/reducing-relocation-stress/ and
https://www.socialworktoday.com/archive/011915p10.shtml.
✓ Aim to build your patient’s resilience by encouraging social interaction and positive thinking. Improved resilience is associated with a healthy lifestyle including good diet and exercise and social engagement: https://www.ageuk.org.uk/globalassets/age-uk/documents/reports-and-publications/reports-and-briefings/health--wellbeing/rb_april15_vulnerability_resilience_improving_later_life.pdf.
✓ Direct informal carers / family members to support networks as they adjust to handing over care of their relative to the care home staff:
https://carers.org/;
https://www.usa.gov/disability-caregiver and https://www.griswoldhomecare.com/blog/relocation-stress-syndrome-checklist-for-moving-elderly-parents/.

## Supplementary Material

cmz014_suppl_Supplementary_Material

## Declaration

Funding: We thank all the past CC75C sponsors for financial support spanning the decades since the Medical Research Council enabled the first follow-up survey (see website for full list of grants: http://www.cc75c.group.cam.ac.uk/background/grants/). A BUPA Foundation grant supported data collection and Abbeyfield Research Foundation funded analysis for this paper. The Wellcome Trust funded CC75C’s collaboration in a theatre production set in a care home supporting wider engagement with this research topic. CC75C was a member study of the National Institute for Health Research funded Collaboration for Leadership in Applied Health Research & Care (CLAHRC) for Cambridgeshire and Peterborough. No funder played any role in the study design; collection, analysis, and interpretation of data; or in writing the report or the decision to submit the article for publication. All researchers were independent from funders.

Ethical approval: Each CC75C study phase was approved by Cambridge Research Ethics Committee (reference numbers: 06_Q0108_87 and 08_H0308_3) and participants’ and proxies’ consent was obtained for each new survey and for the informant interviews after participants had died.
