# Supplementary material for: The experience of transitions in care in very old age: implications for general practice
Source: Fam Pract. 2019 May 4;36(6):778–84. doi: 10.1093/fampra/cmz014 (PMC6859521; doi:10.1093/fampra/cmz014)

Supplementary Data Table
S1 Summary descriptors for CC75C study participants quoted (in order quoted in text)

Name (pseudonym)	Age	Sex	Disability†	Cognitive impairment‡	Moving	Interviews
Stella Thatcher	96	woman	BADLs +IADLs	severe	Moved in with family	Separate: participant + proxy
Rose Baker	98	woman	IADLs only	not cognitively impaired	Moved to sheltered housing, later moved into care after hospital, then to a 2nd care home	Separate: participant + proxy
Patricia Miller	100	woman	BADLs +IADLs	severe	Moved from sheltered housing to a care home near her family	Separate: participant + proxy
Charlotte Smith	98	woman	IADLs only	moderate	Moved in with her family later after hospital moved into a care home	Joint: participant + proxy
Florence Potter	98	woman	BADLs +IADLs	severe	Moved to a care home near family then moved to a 2nd care home	Separate: participant + proxy
Prudence Sawyer	97	woman	BADLs +IADLs	severe	Already moved from sheltered housing to a care home near family after hospital	Proxy informant only: proxy
Hyacinth Fletcher	98	woman	BADLs +IADLs	severe	Moved after fall and hospitalisation from sheltered housing into a care home	Separate: participant + proxy
Margaret Butcher	97	woman	BADLs +IADLs	severe	Moved into a care home as her dementia worsened	Separate: participant + two proxy interviews
Nancy Dempster	97	woman	BADLs +IADLs	mild	Moved into care after falls and care difficulties at home	Proxy informant only
Agatha Cooper	98	woman	BADLs +IADLs	Moderate	Moved into care home as dementia worsened	Separate: participant + proxy
Flora Chamberlain	97	woman	BADLs +IADLs	severe	Moved from living with her family into care after a fall and hospital stay with pneumonia	Separate: participant + proxy
Archibald Faulkner	100	man	BADLs +IADLs	not cognitively impaired	Moved into a care home after fall at home	Separate: participant + proxy
Primrose Turner	98	woman	BADLs +IADLs	moderate	Moved into a care home after repeated falls	Separate: participant + proxy

Loretta Fowler	97	woman	BADLs +IADLs	severe	Already moved to a care home after husband died, dementia	Separate: participant + proxy

Notes for Supplementary data S1

† Mini-Mental State Examination complete scores, plus score category imputation and dementia status if incomplete, categorised 0-17 severe cognitive impairment, 18-21 moderate cognitive impairment, 22-25 mild cognitive impairment, 26-30 normal cognition.
‡ IADL = Instrumental Activities of Daily Living;
BADL = Basic (personal) Activities of Daily Living

Supplementary Data Figure 2 Qualitative Data Sources in CC75C analysed by categories of cognitive function (n=26 aged 95-100 years old) at year 21 (2006-2007)

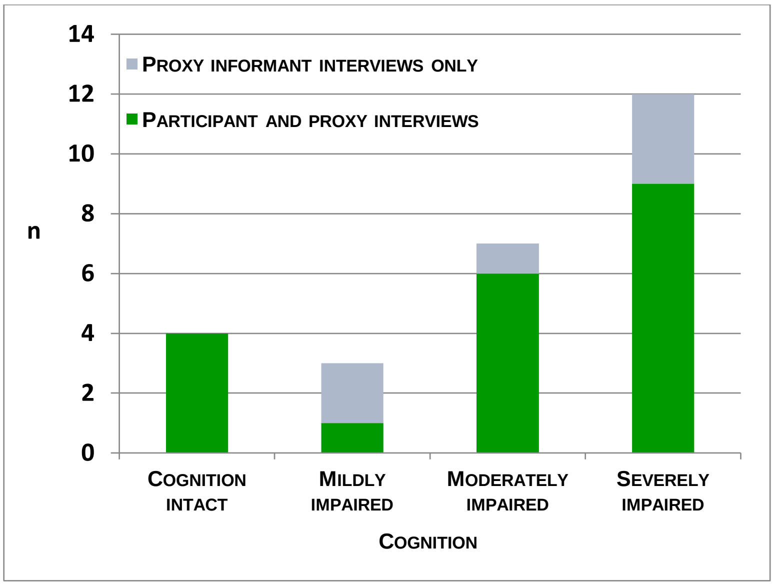

Supplement: cmz014_suppl_Supplementary_Material [file cmz014_suppl_supplementary_material.pdf]
